# Evaluation of the relationship between *ACE2 G8790A* and *AT2R A1675G* gene polymorphisms in COVID-19 patients with and without lung involvement

**DOI:** 10.2478/abm-2024-0022

**Published:** 2024-09-20

**Authors:** Raziye Akcilar, Fatma Emel Kocak, Fatih Kar, Ozben Ozden Isiklar, Sahinde Atlanoglu, Ozlem Genc, Fatima Yaman

**Affiliations:** Department of Physiology, Kütahya Health Sciences University, Faculty of Medicine, Kutahya 43100, Turkey; Department of Medical Biochemistry, Kütahya Health Sciences University, Faculty of Medicine, Kutahya 43100, Turkey; Department of Basic Sciences, Kütahya Health Sciences University, Faculty of Natural and Engineering Sciences, Kutahya 43100, Turkey; Department of Radiology, Kütahya Health Sciences University, Faculty of Medicine, Kutahya 43100, Turkey; Department of Medical Microbiology, Kütahya Health Sciences University, Faculty of Medicine, Kutahya 43100, Turkey; Department of Physical Medicine and Rehabilitation, Kütahya Health Sciences University, Faculty of Medicine, Kutahya 43100, Turkey

**Keywords:** *ACE2 G8790A*, *AT2R A1675G*, COVID-19, gene polymorphism, lung involvement

## Abstract

**Background:**

The SARS-CoV-2 virus produces severe acute respiratory syndrome. The severity of coronavirus disease 2019 (COVID-19) infection is determined by a number of factors, including inherited ones.

**Objectives:**

Our goal is to investigate the link between *ACE2 G8790A* (rs2285666) and *AT2R A1675G* (rs14035430) gene polymorphisms in COVID-19 patients with and without lung involvement.

**Methods:**

A total of 160 COVID-19 patients were divided into 2 groups based on their clinical symptoms: those without lung involvement (control group) and those with lung involvement (infected group). The *ACE2 G8790A* and *AT2R A1675G* gene polymorphisms were analyzed using the PCR-RFLP methods.

**Results:**

The GG genotype, G allele of *ACE2 G8790A*, and GG genotype of *AT2R A1675G* were significantly higher in the control group and had a protective effect against COVID-19 as well as decreased the development of lung involvement (OR = 0.29, 95% CI = 0.10–0.84; OR = 0.40, 95% CI = 0.22–0.72; and OR = 0.33, 95% CI = 0.14–0.78, respectively). Moreover, we found that the AA genotype, A allele of *ACE2 G8790A*, and AG genotype of *AT2R A1675G* increased the risk of COVID-19 in the infected group (OR = 3.50, 95% CI = 1.18–10.3; OR = 2.49, 95% CI = 1.39–4.48; and OR = 3.08, 95% CI = 1.28–7.38, respectively).

**Conclusions:**

These results revealed that a greater frequency of COVID-19 lung involvement in the Turkish population was connected with the AA genotype, the A allele of *ACE2 G8790A*, and the AG genotype of *AT2R A1675G*.

Angiotensin-converting enzyme 2 (ACE2) is an enzyme that is connected to cell membranes and is present in the lungs, arteries, heart, kidneys, and intestines. ACE2 is a type I transmembrane glycoprotein of 805 amino acids with a molecular weight of roughly 120 kDa and an extracellular catalytic domain. The *ACE2* gene, which contains 18 exons and 20 introns, is 39.98 kB long and is located on chromosome Xp22 [1, 2]. ACE2 catalyzes the direct conversion of angiotensin 1–7 from angiotensin II and angiotensin 1–9 from angiotensin I. ACE2 raises angiotensin 1–7 and angiotensin 1–9 levels while decreasing angiotensin I and II substrates [3, 4]. As a result, ACE2 acts as a regulator of the renin–angiotensin–aldosterone system in metabolism, and it could have an effect on cardiovascular and renal function, as well as fertility [5, 6].

Angiotensin II communicates by way of the angiotensin II type 1 and 2 receptors (AT1R and AT2R). The therapeutic actions of AT2R on natriuresis, vasorelaxation, inflammation, wound healing, and tissue remodeling lead to antihypertensive, antiobesity, and organ-protecting effects [7, 8]. AT2R's molecular structure is similar to that of the G protein-coupled receptor superfamily, which has 7 transmembrane domains [9, 10]. The *AT2R* gene, which spans around 5 kb on the X chromosome, contains a polymorphism known as A1675G that is found in intron 1 close to the critical region for the activity of gene transcription [11].

The coronavirus-2, which produces severe acute respiratory illness, is the cause of an emerging global pandemic known as coronavirus disease 2019 (COVID-19). Near the end of 2019, Wuhan, China, was where it was first identified; since then, it has quickly spread over the world. COVID-19 causes pneumonia and acute respiratory distress syndrome (ARDS). It can also cause acute liver, heart, and kidney damage, as well as secondary infections and inflammatory reactions [12]. SARS-CoV-2 is capable of entering lung cells by membrane fusion and endocytosis; when it binds to the ACE2 receptor, the enzyme's activity is reduced [13]. The inflammatory response to the virus is enhanced by reduced ACE2 membrane expression. Additionally, COVID-19 infection raises the levels of angiotensin I and II, while decreasing the levels of angiotensin 1–7 and 1–9 result in decreased activation of Mas, AT2R, and lack of Mas-induced increases in AT2R expression. Alveolar cell survival is reduced by AT1R stimulation and low AT2R expression. It results in inflammation and a rise in vascular permeability [14]. Edema occurs in the alveoli as a result, which inhibits gas exchange and lowers oxygen levels. When all these are considered, acute respiratory distress is exacerbated [15].

Despite the fact that studies have been conducted to look into the relationships between *ACE2* gene polymorphisms and COVID-19, the outcomes of epidemiological studies in various ethnic groups vary. This study also reports the link between COVID-19 and *AT2R A1675G* gene polymorphism for the first time. The goal of this study is to see if there is a link between illness risk, etiology, clinical-laboratory characteristics, lung involvement development, and the *ACE2 G8790A* and *AT2R A1675G* gene polymorphisms in people with COVID-19 who are newly diagnosed, untreated, and without a history of autoimmune disease in the Turkish population.

## Methods

### Study population

The current study was carried out in the Departments of Physiology, Immunology, and Medical Biochemistry at Kütahya Health Sciences University in Kütahya City, Turkey, between January 2021 and June 2021 (after the second wave of the pandemic). The Local Ethics Committee approved this study and all experimental techniques (No: 2020-07/05) of Kütahya Health Sciences University, Kütahya, Turkey and by the Turkish Ministry of Health (2020-09-26T19-21-26). This study was conducted in compliance with the Declaration of Helsinki principles and reported following the recommendation of the Genetic Association Studies (STREGA) an extension of the STROBE Statement [16]. A written informed consent form was signed by each participant.

All of the patients were of Turkish origin from the region of Turkey. We collected the clinical data of 160 patients who applied to the pandemic polyclinic for the first time due to COVID-19. The patients were divided into 2 groups based on disease severity, according to the World Health Organization interim guidance for clinical management of COVID-19 patients [17]. Control group: 80 COVID-19 outpatients who were defined as having no lung involvement, with no abnormal radiological findings, and whose clinical symptoms were mild and the infected group: 80 patients with COVID-19 who were hospitalized for persistent fever, pneumonia, or respiratory distress as detected by chest computed tomography.

The inclusion criteria for this study are: (1) those who had a positive COVID-19 test and were diagnosed for the first time; (2) those who had typical findings for COVID-19 lung involvement (such as respiratory distress, an increase in respiratory rate, a decrease in partial pressure of oxygen in arterial blood at rest, and oxygen saturation in room air); and (3) those who had never received previous COVID-19 treatment.

The exclusion criteria for this study are: (1) those who had a negative RT-PCR test; (2) those who had been smokers; (3) those under the age of 18 years and over the age of 80 years; (4) those who were taking ACE inhibitors and/or AT receptor blockers for hypertension; (5) those with chronic autoimmune diseases such as cardiovascular, thyroid, obstructive lung symptoms, diabetes mellitus, nephropathy, and bronchial asthma; (6) mothers who are pregnant or nursing; and (7) those who began receiving treatment.

### COVID-19 PCR testing

Bio-speedy SARS-CoV-2 RT-PCR detection kit (Bioeksen, Catalog No. BS-SY-WCOR-500) test was used to determine COVID-19 positivity from nasal and pharyngeal swab samples taken from patients by using CFX96 Touch Real-Time PCR Detection System (Bio-Rad).

### Thoracic computed tomography (thoracic CT)

Patients with unusual pneumonia features on a chest X-ray and/or those who had clinical signs of pneumonia, such as dyspnea, tachypnea, coughing, and low oxygen saturation (PO_2_ <92%), had a diagnostic thoracic CT scan [18]. Thoracic CT scans were done in the supine position without the use of an intravenous contrast agent during the inspiration phase using a 16-slice multidetector CT scanner (Aquilion, Toshiba Medical Systems). Scanning parameters are as follows: 120 kVp, averaging 120 mAs (70–250 mAs), 370 FOV, and a section thickness of 3 mm. One millimeter reconstruction images were created from 3 mm images. Images were taken using window settings that made the lung parenchyma visible (window-level [WL], −550 HU; window-width [WW], 1600 HU) and mediastinum (WL, 40 HU; WW, 400 HU). CT images were analyzed on a 2048 × 2560 resolution digital screen.

A radiologist who was blinded to the patients' clinical status reviewed each CT scan. The study comprised patients who had the classic COVID-19 pneumonia symptoms (peripheral, bilateral ground glass-consolidation densities; multifocal round ground glass densities; and inverted halo sign) [19, 20]. Any of these CT findings could have indicated COVID-19 lung involvement in the patients.

### DNA isolation and sample preparation

The individuals' venous blood was obtained in 4 mL and transferred to EDTA-coated tubes. The DNA isolation of blood samples from both groups was done using the usual phenol–chloroform procedure. The DNA's purity was also evaluated on a 0.7% agarose gel electrophoresis and it was kept at – 20°C until further analysis.

### *ACE2 G8790A* (rs2285666) and *AT2R A1675G* (rs14035430) gene polymorphisms

**Figure 1.**
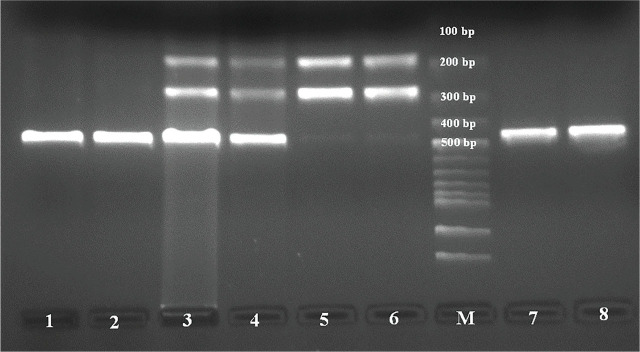
Electrophoresis of *ACE2 G8709A* (rs2285666) gene polymorphism by enzyme digestion product sizes were 466 bp for GG genotype, 185 bp–281 bp–466 bp for GA genotype, and 185 bp–281 bp for AA genotype. Lanes 1 and 2 are the GG genotype; lanes 3 and 4 are the GA genotype; lanes 5 and 6 are the AA genotype; lanes 7 and 8 are the PCR products (466 bp). ACE2, angiotensin-converting enzyme 2; M, DNA molecular weight marker.

**Figure 2.**
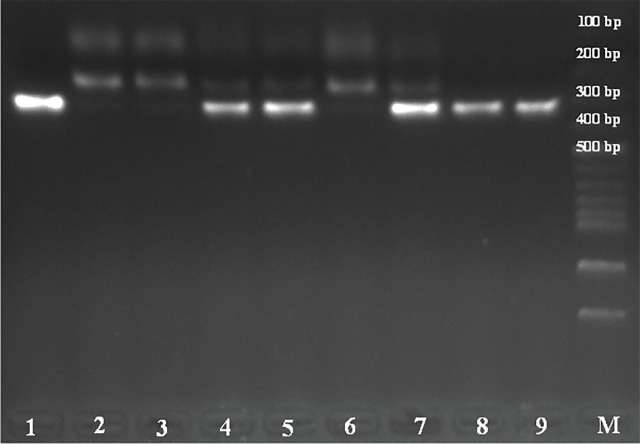
Electrophoresis of *AT2R A1675G* (rs14035430) gene polymorphism by enzyme digestion product sizes were 310 bp for AA genotype, 104 bp–206 bp–310 bp for AG genotype, and 104 bp–206 bp for GG genotype. Lanes 1, 8, and 9 are the AA genotype; lanes 4, 5, and 7 are the AG genotype; lanes 2, 3, and 6 are the GG genotype. AT2R, angiotensin II type 2 receptor; M, DNA molecular weight marker.

To assess *ACE2 G8790A* and *AT2R A1675G* gene polymorphisms, we used the PCR-RFLP technique. Primers' sequences for each SNPs and the PCR conditions are listed in **Table 1**. The PCR products were digested overnight with 5U of Alu1 (*ACE2 G8790A*) and HYP 188 III (*AT2R A1675G*) restriction endonucleases (New England Biolabs, Catalog No. R0137S and R0622S, respectively). The digestion products were separated by 2% agarose gels (BioShop, Catalog No. AGA003.500) stained with ethidium bromide (BioShop, Catalog No. ETB444.50) using 100 bp DNA marker (abm, Catalog No. G193) under ultraviolet (UV) light. The *ACE2 G8790A* and the *AT2R A1675G* genes were genotyped based on the bands that appeared (**Figures 1 and 2**).

**Table 1.** Summary of conditions for the *ACE2 G8790A* and *AT2R A1675G* genetic analyses

	***ACE2 G8790A* (rs2285666)**	***AT2R A1675G* (rs14035430)**
Primer sequence (5′–3′)	F:CATGTGGTCAAAAGGATATCT	F:AGAGATCTGGTGCTATTACG
	R:AAAGTAAGGTTGGCAGACAT	R:CACTTGAAGACTTACTGGTTG
PCR reaction conditions	94°C for 10 min 10 cycles of 94°C for 1 min, 65°C for 1 min, 72°C for 1 min 15 cycles of 94°C for 1 min, 60°C for 1 min, 72°C for 1 min 20 cycles of 94°C for 1 min, 58°C for 1 min, 72°C for 1 min 72°C for 10 min.	95°C for 5 min 35 cycles of 94°C for 45 s, 55°C for 1 min, 72°C for 1 min 72°C for 7 min.
PCR product size	466 bp	310 bp
Restriction enzyme, incubation conditions	Alu I	HYP 188 III
	37°C overnight	37°C overnight
Fragment length (bp)	GG: 466 bp	AA: 310 bp
	GA: 185 bp–281 bp–466 bp	GA: 104 bp–206 bp–310 bp
	AA: 185 bp–281 bp	GG: 104 bp–206 bp

ACE2, angiotensin-converting enzyme 2; AT2R, angiotensin II type 2 receptor.

### Measurement of clinical data and biochemical indicators

Venous blood samples were collected in a non-heparinized tube for analysis of biochemical parameters. All blood samples were centrifuged at 3000 *g* for 10 min at room temperature within 1 h.

The following laboratory tests were routinely performed on patients admitted to the COVID polyclinic: The automated hematology analyzer (Mindray Bio-Medical Electronics Co., Ltd.) with original reagents (Catalog No: 161151224) was used to analyze whole blood counts. Serum aspartate aminotransferase (AST), alanine aminotransferase (ALT) (Catalog No. OSR6209, OSR6107, respectively), urea, creatinine, C-reactive protein (CRP) levels (Catalog No. OSR6234, OSR6178, OSR6199, respectively), and serum ferritin (Catalog No. 33020) levels were measured with Beckman Coulter AU5800 analyzer and UniCel^®^ DxI 800 immunoassay system (Beckman Coulter), respectively. Fibrinogen and D-dimer levels (Catalog No: 53906, 01003, respectively) were determined using Sysmex^®^ CS-5100 SystemTM coagulation analyzer (Siemens Healthcare Diagnostics), respectively.

### Statistical analysis

The SPSS 20 program (SPSS Inc.) was used to conduct all statistical analyses. The genotype frequencies of the patients and controls were compared using the χ^2^-test to check if there were any differences. A Student's *t*-test was used to test continuous data, which was provided as the mean standard deviation (SD). The correlation values of clinical parameters between control and infected groups with *ACE2 G8709A* and *AT2R A1675G* gene polymorphisms were compared using One-Way Analysis of Variance (ANOVA). For genetic equilibrium tests, the Hardy–Weinberg equilibrium was used. The odds ratio (OR) within 95% confidence intervals (CIs) was calculated using logistic regression analysis. Statistical significance was determined at the level of *P*-value ≤0.05.

The size of the sample used in this study was calculated through the GPower 3.1 software (Düsseldorf, Germany). Since student's *t* and χ^2^ tests will be used for the statistical analysis of the data, these tests were chosen on the GPower. In general, the type I error value α is 0.05 and the type II error value β is 0.05, so the power value of the study is determined as 0.86, which was made at the 2.49 OR and 0.05 significance level (95% Cl). As a result of the power analysis performed under these conditions, the total sample size was determined as 160, with 80 observations in each group.

## Results

### Patient characteristics and clinical features of COVID-19

Patient characteristics and clinical features of 160 patients with COVID-19 are summarized in **Table 2**. The study group consisted of 80 COVID-19 patients without lung involvement (control group) (42 men and 38 women; mean age: 41.3 ± 16.0 SD years) and 80 COVID-19 patients with lung involvement (infected group) (39 men and 41 women; mean age: 48.3 ± 16.7 SD years). Higher levels of white blood cell (WBC), neutrophil, D-dimer, CRP, urea, and ferritin were determined in the infected group. Although the fibrinogen level was increased, it was not statistically significant in the same patient group. In addition, there was no statistically significant difference in the other parameters between the 2 groups (**Table 2**).

**Table 2.** Characteristics of the study population

**Variables**	**Control Group n = 80**	**Infected Group n = 80**	***P*-value**
Sex (M/F) n (%)	42 (52.5)/38 (47.5)	39 (48.8)/41 (51.2)	0.63
Age (years)	41.3 ± 16.0	48.3 ± 16.7	0.20
WBC (10^3^/mm^3^)	6.47 ± 2.14	6.96 ± 3.26	0.008^**^
PLT (10^3^/mm^3^)	226.1 ± 63.4	213.0 ± 57.4	0.44
Neutrophil (10^3^/μL)	4.21 ± 1.93	4.84 ± 2.80	0.023^*^
Lymphocyte (10^3^/μL)	1.64 ± 0.78	1.60 ± 0.80	0.45
Eosinophil (10^3^/μL)	00.7 ± 0.08	0.05 ± 0.10	0.65
Hemoglobin (g/dL)	14.4 ± 1.87	13.9 ± 2.42	0.40
D-dimer (μg/mL)	510.0 ± 391.9	825.7 ± 818.3	<0.001^**^
Fibrinogen (mg/dL)	340.1 ± 122.6	429.9 ± 126.2	0.07
Ferritin (ng/mL)	91.4 ± 123.8	253.3 ± 323.4	<0.001^**^
CRP (mg/L)	19.1 ± 35.6	55.3 ± 67.3	<0.001^**^
AST (IU/L)	27.6 ± 17.0	32.3 ± 15.8	0.25
ALT (IU/L)	29.3 ± 26.3	29.5 ± 15.5	0.34
Creatinin (mg/dL)	0.96 ± 0.18	1.04 ± 0.57	0.27
Urea (mg/dL)	28.1 ± 11.4	34.4 ± 22.3	0.018^*^

Analyzed using student's *t*-test or chi-square (χ^2^) test.

Values are presented as mean ± SD or n (%).

ALT, alanine aminotransferase; AST, aspartate aminotransferase; CRP, C-reactive protein; PLT, platelet; SD, standard deviation; WBC, white blood cell.

* *P* ≤ 0.05,

** *P* < 0.01.

### Analyses of the *ACE2 G8790A* and *AT2R A1675G* gene polymorphisms

The frequency of *ACE2 G8790A* gene polymorphism genotypes in the control group and the infected group showed a significant deviation from Hardy–Weinberg equilibrium (*P* < 0.05) (**Table 3**). The observed and expected frequencies of the *AT2R A1675G* gene polymorphism in the infected group were in Hardy–Weinberg equilibrium, whereas in the control group, it was not (**Table 3**).

**Table 3.** Hardy–Weinberg equilibrium for *ACE2 G8790A* and *AT2R A1675G* gene polymorphisms in COVID patients without/with lung involvement

**Genotype**	**Observed**	**Expected**	**χ^2^**	***P*-value**	**Alleles**	**Frequency**
***ACE2***						

**Control group**						
GG	65	61.3	14.6	<0.001^**^	G	0.13
GA	10	17.5			A	0.87
AA	5	1.3				
**Infected group**						
GG	52	43.5	24	<0.001^**^	G	0.26
GA	14	31			A	0.74
AA	14	5.5				

***AT2R***						

**Control group**						
AA	37	26.5	23.2	<0.001^**^	A	0.43
AG	18	39.1			G	0.57
GG	25	14.5				
**Infected group**						
AA	35	31.9	2.24	0.133	A	0.37
AG	31	37.2			G	0.63
GG	14	10.9				

Data were analyzed by chi-square (χ^2^) test.

ACE2, angiotensin-converting enzyme 2; AT2R, angiotensin II type 2 receptor; COVID-19, coronavirus diseases 2019; *P*, probability of difference.

** *P* < 0.01.

**Table 4** summarizes the allele frequency and genotype distribution of the *ACE2 G8790A* and *AT2R A1675G* gene polymorphisms in the control group and the infected group. Significant differences were found in the genotype and allele frequency of the *ACE2 G8790A* gene polymorphism between the 2 groups (χ^2^ = 6.37, df = 2, *P* = 0.04 and χ^2^ = 9.68, df = 1, *P* = 0.002, respectively). In the control group, 65 GG (81.2%), 10 GA (12.5%), and 5 AA (6.2%) genotypes were found, while the genotypes were 52 (65%), 14 (17.5%), and 14 (17.5%) in the infected group, respectively. The G allele was encountered in 87.5% (140) of the control group and 73.8% (118) of the infected group. The A allele was seen in 12.5% (20) of the control group and 26.2% (42) of the moderate/severe disease. Our results showed that both the AA genotype and the A allele frequencies were increased in the infected group compared with the control group (*P* = 0.04 and *P* = 0.002, respectively), indicating that the AA genotype and the A allele were associated with an increased risk of developing of lung involvement in the infected group (OR = 3.50, 95% CI = 1.18–10.3; OR = 2.49, 95% CI = 1.39–4.48). The GG genotype and G allele were significantly higher in the control group and had a protective effect against COVID-19 and decreased the lung involvement development (OR = 0.29, 95% CI = 0.10–0.84; OR = 0.40, 95% CI = 0.22–0.72).

**Table 4.** Distribution of *ACE2 (G8709A)* and *AT2R (A1675G)* genotypes and alleles frequencies in COVID patients without/with lung involvement

		**Control Group**		**Infected Group**		**OR (95% CI)**	***P*-value**	**OR (95% CI)**	***P*-value**
		
		**n = 80**	**%**	**n = 80**	**%**				
	GG	65	55.6	52	44.4	1	-	0.29 (0.10–0.84)	0.01^*^
	GA	10	41.7	14	58.3	1.75 (0.72–4.26)	0.21	0.50 (0.14–1.84)	0.29
	AA	5	26.3	14	73.7	3.50 (1.18–10.3)	0.001^**^	1	-
*ACE2* *G8709A* (rs2285666)	χ^2^ = 6.37, df = 2, *P* = 0.04^*^								
	G allele	140	54.3	118	45.7	1	-	0.40 (0.22–0.72)	0.001^**^
	A allele	20	32.3	42	67.7	2.49 (1.39–4.48)	0.001^**^	1	-
	χ^2^ = 9.68, df = 1, *P* = 0.002^**^								
	AA	37	51.4	35	48.6	1.69 (0.76–3.76)	0.19	0.55 (0.26–1.15)	0.11
	AG	18	36.7	31	63.3	3.08 (1.28–7.38)	0.01^*^	1	
	GG	25	64.1	14	35.9	1	0.33 (0.14–0.78)	0.01^*^	
*AT2R* *A1675G* (rs14035430)	χ^2^ = 6.60, df = 2, *P* = 0.03^*^								
	A allele	92	47.7	101	52.3	1.27 (0.81–1.98)	0.30	1	
	G allele	68	53.5	59	46.5	1	0.79 (0.50–1.24)	0.30	
	χ^2^ = 1.05, df = 1, *P* = 0.30								

Data were analyzed by chi-square test.

ACE2, angiotensin-converting enzyme 2; AT2R, angiotensin II type 2 receptor; CI, confidence interval; COVID-19, coronavirus diseases 2019; OR, odds ratio; *P*, probability of difference.

* *P* < 0.05,

** *P* < 0.01.

Significant differences were found in the genotype frequencies of the *AT2R A1675G* gene polymorphism between the 2 groups (χ^2^ = 6.60, df = 2, *P* = 0.03). The frequencies of the AA, AG, and GG genotypes of *AT2R A1675G* gene polymorphism in the control group were 51.4%, 36.7%, and 64.1% and in the infected group were 48.6%, 63.3%, and 35.9%, respectively. The frequency of the AG genotype was higher in the infected group, whereas the frequency of the GG genotype was higher in the control group. The risk of developing COVID-19 in individuals with the AG genotype is 3.08 times higher than in individuals with the GG genotype (OR = 3.08, 95% CI = 1.28–7.38, *P* = 0.01). The risk of developing COVID-19 in individuals with the GG genotype is 0.33 times less than in individuals with the AG genotype (OR = 0.33, 95% CI = 0.14–0.78, *P* = 0.01) (**Table 3**). There was no significant difference in allele frequencies of the *AT2R A1675G* gene polymorphism between the 2 groups (χ^2^ = 1.05, df = 1, *P* = 0.30). The frequencies of the A and G alleles of the *AT2R A1675G* gene polymorphism in the control group were 47.7% and 53.5%, in the infected group were 52.3% and 46.5%, respectively (**Table 4**).

### Influence of the *ACE2 G8790A* and *AT2R A1675G* gene polymorphisms on clinical-laboratory variables in COVID-19 patients without lung involvement

Serum D-dimer and fibrinogen levels were 510.0 ± 391.9 μg/mL and 340.1 ± 122.6 mg/dL in the control group. When we compared the D-dimer, fibrinogen levels, and *ACE2 G8790A* gene polymorphism, D-dimer and fibrinogen levels of the COVID-19 patients without lung involvement with GG genotype were 988.3 ± 941.2 μg/mL and 455.9 ± 139.1 mg/dL, with GA genotype were 534.5 ± 386.6 μg/mL and 384.2 ± 97.9 mg/dL, and with AA genotype were 513.1 ± 376.7 μg/mL, 379.2 ± 59.0 mg/dL, respectively. Serum D-dimer and fibrinogen levels of the COVID-19 patients without lung involvement with the GG genotype were higher (*P* = 0.05 and *P* = 0.04). Although other clinical-laboratory variables (age, WBC, etc.) of the COVID-19 patients without lung involvement with GG genotype were higher, there were no significant differences. The GG genotype may decrease the lung involvement development in the control group of COVID-19 patients (**Table 5**).

**Table 5.** Analysis of the influence of *ACE2 G8790A* and *AT2R A1675G* gene polymorphisms on clinical-laboratory variables in COVID-19 patients without lung involvement

**Variables**	**Control Group (n = 80)**							

	***ACE2 G8709A* genotype**				***AT2R A1675G* genotype**			

	**GG**	**GA**	**AA**	***P*-value**	**AA**	**AG**	**GG**	***P*-value**
Age (years)	49.9 ± 16.7	43.0 ± 17.6	48.0 ± 15.8	0.39	49.9 ± 17.2	45.3 ± 18.9	51.4 ± 7.90	0.43
WBC (10^3^/mm^3^)	7.21 ± 3.27	5.98 ± 2.02	6.98 ± 4.18	0.46	6.65 ± 2.50	7.06 ± 3.79	7.49 ± 3.83	0.70
PLT (10^3^/mm^3^)	213.2 ± 61.0	214.6 ± 48.2	210.5 ± 55.2	0.98	208.4 ± 55.3	217.6 ± 59.4	214.4 ± 61.0	0.80
Neutrophil (10^3^/μL)	5.08 ± 2.81	3.70 ± 1.68	5.06 ± 3.52	0.25	4.71 ± 2.39	4.74 ± 3.06	5.38 ± 3.31	0.73
Lymphocyte (10^3^/μL)	1.61 ± 0.84	1.78 ± 0.70	1.36 ± 0.71	0.39	1.45 ± 0.71	1.79 ± 0.98	1.55 ± 0.45	0.21
Eosinophil (10^3^/μL)	00.6 ± 0.12	0.04 ± 0.04	0.06 ± 0.09	0.82	0.05 ± 0.08	0.07 ± 0.14	0.04 ± 0.07	0.62
Hemoglobin (g/dL)	13.6 ± 2.70	14.4 ± 1.78	14.2 ± 1.75	0.47	13.6 ± 3.03	13.9 ± 1.88	14.3 ± 1.68	0.61
D-dimer (μg/mL)	988.3 ± 941.2	534.5 ± 386.6	513.1 ± 376.7	0.05^*^	914.5 ± 1044.5	784.0 ± 381.8	696.2 ± 914.2	0.66
Fibrinogen (mg/dL)	455.9 ± 139.1	384.2 ± 97.9	379.2 ± 59.0	0.04^*^	442.1 ± 131.5	425.7 ± 129.4	408.7 ± 109.4	0.69
Ferritin (ng/mL)	289.3 ± 374.0	164.2 ± 133.4	208.5 ± 230.1	0.37	297.9 ± 362.6	148.0 ± 179.0	374.7 ± 412.3	0.05^*^
CRP (mg/L)	68.2 ± 76.6	32.1 ± 38.4	30.6 ± 32.7	0.06	67.4 ± 80.8	37.3 ± 44.8	65.0 ± 67.1	0.16
AST (IU/L)	34.0 ± 17.3	30.7 ± 13.9	27.1 ± 10.7	0.32	29.9 ± 14.1	29.3 ± 9.42	44.6 ± 24.6	0.005^**^
ALT (IU/L)	31.7 ± 16.9	26.9 ± 12.2	23.6 ± 10.9	0.17	27.8 ± 16.1	26.0 ± 12.2	41.2 ± 15.7	0.005^**^
Creatinin (mg/dL)	1.09 ± 0.69	0.96 ± 0.18	0.97 ± 0.27	0.67	1.16 ± 0.83	0.93 ± 0.21	0.99 ± 0.13	0.25
Urea (mg/dL)	36.6 ± 25.3	31.8 ± 17.3	28.7 ± 12.0	0.45	39.5 ± 29.9	30.1 ± 13.8	30.7 ± 10.8	0.19

Data were analyzed by ANOVA.

Values are presented as mean ± SD.

ACE2, angiotensin-converting enzyme 2; ALT, alanine aminotransferase; AST, aspartate aminotransferase; AT2R, angiotensin II type 2 receptor; COVID-19, coronavirus diseases 2019; CRP, C-reactive protein; *P*, probability of difference; PLT, platelet; SD, standard deviation; WBC, white blood cell.

* *P* ≤ 0.05,

** *P* < 0.01.

When we compared the ferritin, AST, ALT levels, and *AT2R A1675G* gene polymorphism, ferritin, AST, and ALT levels of the COVID-19 patients without lung involvement with GG genotype were 374.7 ± 412.3 ng/mL, 44.6 ± 24.6 IU/L, and 41.2 ± 15.7 IU/L, with AG genotype were 148.0 ± 179.0 ng/mL, 29.3 ± 9.42 IU/L, and 26.0 ± 12.2 IU/L, and with AA genotype were 297.9 ± 362.6 ng/mL, 29.9 ± 14.1 IU/L, and 27.8 ± 16.1, respectively. Serum ferritin, AST, and ALT levels of the COVID-19 patients without lung involvement with the GG genotype were higher (*P* = 0.05, *P* = 0.005, and *P* = 0.005, respectively) (**Table 5**).

### Influence of the *ACE2 G8790A* and *AT2R A1675G* gene polymorphism on clinical-laboratory variables in COVID-19 patients with lung involvement

When we compared the clinical-laboratory variables, and *ACE2 G8790A* gene polymorphism, age (51.8 ± 26.1), hemoglobin (15.8 ± 0.92 g/dL), creatinin (1.05 ± 0.15 mg/dL), urea (45.6 ± 28.7 mg/dL) levels of the COVID-19 patients with lung involvement with AA genotype were higher compared with the GG and GA genotypes. Although the D-dimer, Ferritin, CRP, ALT levels of COVID-19 patients with lung involvement with the AA genotype were high, it was not statistically significant (*P* > 0.05). The AA genotype may increase the level of some clinical-laboratory variables, resulting in increased development of lung involvement risk in the infected group of COVID-19 patients (**Table 6**).

**Table 6.** Analysis of the influence of *ACE2 G8790A* and *AT2R A1675G* gene polymorphisms on clinical-laboratory variables in COVID-19 patients with lung involvement

**Variables**	**Infected group (n = 80)**							

	***ACE2 G8709A* genotype**				***AT2R A1675G* genotype**			

	**GG**	**GA**	**AA**	***P*-value**	**AA**	**AG**	**GG**	***P*-value**
Age (years)	42.2 ± 14.7	30.8 ± 14.3	51.8 ± 26.1	0.03^*^	38.4 ± 16.7	44.4 ± 11.8	45.6 ± 17.1	0.22
WBC (10^3^/mm^3^)	6.63 ± 2.16	5.66 ± 1.91	6.00 ± 2.43	0.36	6.49 ± 2.04	6.86 ± 1.94	6.17 ± 2.46	0.58
PLT (10^3^/mm^3^)	228.4 ± 64.6	235.1 ± 51.7	179.0 ± 60.0	0.22	236.2 ± 618.9	228.3 ± 65.4	209.6 ± 51.3	0.26
Neutrophil (10^3^/μL)	4.35 ± 1.90	3.40 ± 1.81	4.08 ± 2.52	0.35	4.16 ± 2.03	4.54 ± 1.80	4.05 ± 1.91	0.69
Lymphocyte (10^3^/μL)	1.64 ± 0.78	1.72 ± 0.61	1.45 ± 1.23	0.83	1.71 ± 0.74	1.64 ± 0.72	1.52 ± 0.89	0.64
Eosinophil (10^3^/μL)	00.8 ± 0.08	0.06 ± 0.04	0.05 ± 0.05	0.53	0.07 ± 0.07	0.07 ± 0.08	0.07 ± 0.09	0.99
Hemoglobin (g/dL)	14.5 ± 1.83	13.2 ± 1.93	15.8 ± 0.92	0.02^*^	14.3 ± 1.71	14.3 ± 2.29	14.6 ± 1.83	0.75
D-dimer (μg/mL)	491.4 ± 349.7	559.1 ± 566.6	654.6 ± 562.9	0.61	414.9 ± 340.8	641.4 ± 483.6	523.1 ± 303.8	0.08
Fibrinogen (mg/dL)	345.1 ± 129.6	312.7 ± 89.7	330.4 ± 87.1	0.73	324.4 ± 129.0	354.9 ± 132.9	352.0 ± 92.8	0.57
Ferritin (ng/mL)	93.6 ± 131.5	45.8 ± 59.0	154.8 ± 89.5	0.26	88.1 ± 144.3	95.4 ± 115.5	93.0 ± 90.8	0.97
CRP (mg/L)	17.9 ± 33.5	15.1 ± 19.7	42.6 ± 73.9	0.31	17.9 ± 40.5	27.0 ± 42.1	15.3 ± 18.7	0.55
AST (IU/L)	28.4 ± 18.6	23.4 ± 6.60	26.6 ± 5.36	0.68	24.9 ± 8.51	30.6 ± 20.0	29.6 ± 23.2	0.40
ALT (IU/L)	30.1 ± 27.7	20.7 ± 11.3	35.6 ± 29.3	0.49	25.1 ± 15.7	43.0 ± 46.1	25.7 ± 14.9	0.04^*^
Creatinin (mg/dL)	0.98 ± 0.18	0.78 ± 0.12	1.05 ± 0.15	0.002^**^	0.93 ± 0.18	1.02 ± 0.19	0.96 ± 0.18	0.22
Urea (mg/dL)	27.8 ± 8.75	21.5 ± 4.60	45.6 ± 28.7	<0.001^**^	27.6 ± 9.00	26.4 ± 7.08	30.0 ± 16.3	0.56

Data were analyzed by ANOVA.

Values are presented as mean ± SD.

ACE2, angiotensin-converting enzyme 2; ALT, alanine aminotransferase; AST, aspartate aminotransferase; AT2R, angiotensin II type 2 receptor; COVID-19, coronavirus diseases 2019; CRP, C-reactive protein; *P*, probability of difference; PLT, platelet; SD, standard deviation; WBC, white blood cell.

* *P* ≤ 0.05,

** *P* < 0.01.

When we compared the clinical-laboratory variables, and *AT2R A1675G* gene polymorphism, ALT (43.0 ± 46.1 IU/L) level of the COVID-19 patients with lung involvement with AG genotype were higher compared with the GG (25.7 ± 14.9 IU/L) and AA (25.1 ± 15.7 IU/L) genotypes (*P* = 0.04). D-dimer, fibrinogen, ferritin levels of COVID-19 patients with lung involvement with AG genotype were high, it was not statistically significant (*P* > 0.05) (**Table 6**).

## Discussion

COVID-19 has become a worldwide wave of crisis, threatening human health and global economic stability because of its rapid and progressive geographic spread. Infected people may experience symptoms like fever, dry cough, dyspnea, and tiredness [21, 22]. COVID-19 infection causes ARDS and, in severe cases, death [22, 23]. Several investigations have shown that COVID-19 patients had lymphopenia, thrombocytopenia, and leukopenia [24,25,26]. Many patients also had increased levels of D-dimer, serum ferritin, ALT, AST, CRP, lactate dehydrogenase (LDH), creatine kinase (CK), and prolonged prothrombin time in COVID-19 infection [27,28,29,30]. In this study, although the increased fibrinogen levels were not statistically significant, the WBC, neutrophil, D-dimer, CRP, urea, and ferritin levels increased in COVID-19 patients with lung involvement. Our study's findings were similarly consistent with those of previous studies [27,28,29,30].

ACE2 can cleave angiotensin II to angiotensin 1–7, which can decrease inflammation and fibrosis while also causing vasodilation by connecting to the Mas receptor. It is known that the ACE2/angiotensin 1–7/Mas axis has protective effects on the lungs [31]. Imai et al. [32] have found that ACE2 appears to protect against severe lung injury in ACE2 knockout mice. Pulmonary damage with associated respiratory distress is one of the main causes of morbidity and mortality in COVID-19 infection. The S1 domain of the spike protein of SARS-CoV-2 competes with angiotensin II for binding to ACE2. The binding of the S protein to ACE2 blocks ACE2 activity, resulting in ACE/ACE2 imbalance. The ACE/ACE2 imbalance leads to an increase in angiotensin II-mediated vasoconstriction, fibrosis, apoptosis, and damage in alveolar epithelial cells [33]. As a result, patients may experience more severe COVID-19.

The location of the *ACE2 G8790A* (rs2285666) gene polymorphism in the third intron's fourth base and in the intron next to the exon suggests that this region may change mRNA alternative splicing and influence the expression of the ACE2 receptor gene [34].

To explain population-based variations in COVID-19 severity, several studies have looked at *ACE2* polymorphisms [2,3,4,5,6,7,8,9,10,11,12,13,14,15,16,17,18,19,20,21,22,23,24,25,26,27,28,29,30,31,32,33,34,35]. Karakaş Çelik et al. [36] and Novelli et al. [37] reported that the *ACE2 G8790A* gene polymorphism has no effect on COVID-19 severity. According to Malik et al. [38], the *ACE2 G8790A GG* genotype is associated with a significant risk of COVID-19 severity. Möhlendick et al. [39] found that bearers of the *ACE2* rs2285666 GG or G allele had a nearly 2-fold increased risk of SARS-CoV-2 infection and a 3-fold increased risk of serious disease or COVID-19 mortality compared with AA genotypes. Srivastava et al. [40] demonstrated that the A allele has been linked to a reduced risk of infection and death from COVID-19. Asselta et al. [41] investigated that the A allele appears to be less common among severely impacted populations, such as Italians and other Europeans, compared with Asians, who appear to have a higher frequency of the A allele and have demonstrated a better epidemiological status. Gómez et al. [42] have found that the *ACE2* rs2285666 A allele was linked to hypertension in the older population, with no significant difference between mild and severe COVID-19 individuals. In our study, for the *ACE2 G8790A* genotype and allele frequencies, there was a significant difference between COVID-19 patients without and with lung involvement. In COVID-19 patients without lung involvement, the GG genotype and G allele were significantly greater, and the levels of serum D-dimer and fibrinogen in these patients with the GG genotype were increased. The GG genotype of the *ACE2 G8790A* gene had a protective effect against COVID-19 and decreased the lung involvement. Also, we observed that the AA genotype and A allele of *ACE2 G8790A* were associated with a 3.50- and 2.49-fold increase in the risk of COVID-19 compared with the GG genotype and G allele in COVID-19 patients with lung involvement, respectively. In this study and previous studies on this subject, many factors may cause differences in susceptibility to the pathogenesis and severity of COVID-19, including both altered *ACE2 G8790A* gene polymorphism and various comorbidities such as age, gender, ethnicity, drug therapy, and cardiovascular disease and metabolic syndrome.

ACE2 levels in the blood are higher in those with active COVID-19 disease and the days after infection, according to Patel et al. [43]. van Lier et al. [44] discovered that people who had COVID-19 disease risk factors had higher circulating ACE2. In another study, it was determined that in COVID-19, serum ACE2 levels rose as new indicators of severe lung disease with vascular injury [45]. Kornilov et al. [46] showed that higher levels of plasma soluble form ACE2 were linked to men, cardiovascular disease, obesity, diabetes, and older age, all of which are major risk factors for COVID-19 infection complications and mortality. The *ACE2 G8790A* gene polymorphism may influence the conversion of ACE2 total RNA to mRNA as well as the amount of protein produced. One study looked at the effect of the *ACE2 G8790A* gene polymorphism on serum ACE2 levels and discovered that A carriers had considerably greater levels than G allel [47]. It was not possible to look at serum ACE2 concentrations in COVID-19 patients. Therefore, we cannot compare the results of serum ACE2 levels with those of *ACE2 G8790A* gene polymorphism in these patient groups.

In SARS-CoV-2 infection, depletion of ACE2, reduction of angiotensin 1–7, angiotensin 1–9, and as a result, less activation of Mas, AT2R, and Mas-related G-protein-coupled receptor member D (MrgD), and the absence of Mas-induced increases in AT2R expression may result in disruption of the protective arm of the renin-angiotensin system (RAS) [48]. AT2R has anti-inflammatory and anti-fibrotic effects in lung tissue and may be of significance in light of the lung pathology presentation in COVID-19 [49]. The human *AT2R* gene is a 363-amino acid protein with a molecular weight of 41 kDa resides on the X chromosome (q22–q23), and consists of 3 exons and 2 introns [11]. A polymorphism, designated as A1675G lies in intron 1 near to the important region for gene transcription activity. The *AT2R A1675G* gene polymorphism (rs14035430) has been investigated in several diseases [50]. COVID-19 patients with lung involvement in our study had a higher prevalence of AG genotype. AG genotype of *AT2R A1675G* was associated with 3.08-fold increase in the risk of COVID-19 compared with GG genotype. In addition, we observed a marked decrease in COVID-19 and lung involvement risk in carriers of the GG genotype in COVID-19 patients without lung involvement. The relationship between AT2R and COVID-19 was unclear in the literature. In addition, there has not been any study in the literature between *AT2R A1675G* gene polymorphism and COVID-19. This is the first study ascribing an association between *AT2R A1675G* gene polymorphism and COVID-19 patients in the Turkish population. Therefore, it is not possible to compare the results of this study with *AT2R A1675G* gene polymorphism and lung involvement in COVID-19 patients. It is advised to test these findings in more significant and diverse populations in order to show the validity of our findings.

Our research has a few drawbacks. The study only includes a small number of subjects since genotypic analysis is expensive. Second, we did not compare blood ACE2 and angiotensin II levels with the *ACE2 G8790A* and *AT2R A1675G* gene polymorphisms. Further investigations are required to confirm the results of this polymorphism with serum ACE2 and angiotensin II levels.

## Conclusion

We found that AA genotype and A allele of *ACE2 G8790A* and AG genotype of *AT2R A1675G* are linked to an increased risk of COVID-19 infection and lung involvement, respectively. Our results suggest that *ACE2 G8790A* and *AT2R A1675G* gene polymorphisms could be considered genetic and risk factors for the development of COVID-19 in Turkish patients. To fully comprehend the *ACE2 G8790A* gene polymorphism and its clinical significance in COVID-19, large-scale investigations and long-term results in various ethnic groups are needed in other populations to corroborate these findings.
